# Goodness-of-Fit Based Secure Cooperative Spectrum Sensing for Cognitive Radio Network

**DOI:** 10.1155/2014/752507

**Published:** 2014-05-18

**Authors:** Hiep Vu-Van, Insoo Koo

**Affiliations:** The School of Electrical Engineering, University of Ulsan, San 29, Muger 2-dong, Ulsan 680-749, Republic of Korea

## Abstract

Cognitive radio (CR) is a promising technology for improving usage of frequency band. Cognitive radio users (CUs) are allowed to use the bands without interference in operation of licensed users. Reliable sensing information about status of licensed band is a prerequirement for CR network. Cooperative spectrum sensing (CSS) is able to offer an improved sensing reliability compared to individual sensing. However, the sensing performance of CSS can be destroyed due to the appearance of some malicious users. In this paper, we propose a goodness-of-fit (GOF) based cooperative spectrum sensing scheme to detect the dissimilarity between sensing information of normal CUs and that of malicious users, and reject their harmful effect to CSS. The empirical CDF will be used in GOF test to determine the measured distance between distributions of observation sample set according to each hypothesis of licensed user signal. Further, the DS theory is used to combine results of multi-GOF tests. The simulation results demonstrate that the proposed scheme can protect the sensing process against the attack from malicious users.

## 1. Introduction

Nowadays, more bandwidth and higher bit-rates have been required to meet usage demands due to an explosion in wireless communication technology. According to the Federal Communications Commission's spectrum policy task force report [1], the actual utilization of the licensed spectrum varies from 15% to 80%. In some cases, the utilization is only a small percentage of the total capacity. Cognitive radio (CR) technology [2] has been proposed to solve the problem of ineffective utilization of spectrum bands. Both unlicensed and licensed users, termed the cognitive radio user (CU) and primary user (PU), respectively, operate in CR networks. In CR network, CUs are allowed to access the frequency assigned to PU when it is free. But CU must vacate the occupied frequency when the presence of PU is detected. Therefore, reliable detection of the PU's signal is a requirement of CR networks.

In order to ascertain the presence of a PU, CUs can use one of several common detection methods, such as matched filter, feature, and energy detection [2, 3]. Energy detection is the optimal sensing method if the CU has limited information about PU's signal (e.g., only the local noise power is known) [3]. In energy detection, frequency energy in the sensing channel is received in a fixed bandwidth *W* over an observation time window *T* to compare with the energy threshold and determine whether or not the channel is utilized. However, the received signal power may fluctuate severely due to multipath fading and shadowing effects. Therefore, it is difficult to obtain reliable detection with only one CU. Better sensing performance can be obtained by allowing some CUs to perform cooperative spectrum sensing [4–6].

CSS can use some combination methods such as equal gain combination (EGC) and maximum gain combination (MGC) [7] to combine sensing information of all CUs in the network and make a global decision about status of PU signal. Since EGC gives the same weight for all CUs in the network, it is easy to execute but with limited performance. MGC is known as the optimal combination rule. However, it requires information about the SNRs of the sensing channel, which is difficult to obtain in practice. In addition, MGC is sensitive to attack by malicious users who send false sensing data to the fusion center (FC) [8]. The research presented in [8, 9] determined that the presence of a few malicious users can severely reduce the performance of a CSS scheme. Algorithms used to identify the malicious users have been proposed in the studies of [8, 9]. In previous research, a simple technique (i.e., outlier-detection) is used to detect less damage malicious CUs such as* always No* or* always Yes* CU. In addition, the technique is unable to protect the CSS in the event of a large number of malicious users in the network.

In this paper, we utilize multi-goodness-of-fit (GOF) tests to design a robust CSS, in which the event detection technique [10, 11] will be used to provide the combination of different evidence of each type of GOF test which are supported by a particular hypothesis of PU signal. The proposed scheme considers two types of GOF tests, Kolmogorov-Smirnov (KS) and Cramer-von Mises (CM) tests. The proposed scheme can distinguish the sensing information of normal CUs and that of malicious users and reject the harmful effect of malicious user to sensing combination process. Three common types of malicious users including* always Yes*,* always No,* and* opposite* are considered in this paper.

## 2. Background

### 2.1. Goodness-of-Fit Test

The GOF test summarizes the discrepancy between the observed samples with theoretical distributions or empirical distributions and the reference distribution. For the *n* independent and identical distributed observation, the sample is first arranged in ascending order such that *s*_1_ ≤ *s*_2_ ≤ ⋯≤*s*_*n*_. The GOF test is used to determine whether or not the samples set was drawn from the same distribution with a cumulative distribution function (CDF) *F*_0_. The testing hypothesis can be formulated as follows:

(1) $$\begin{array}{c} F\left(s\right)=F_{0}\left(s\right):H_{o},\\ F\left(s\right)\neq F_{0}\left(s\right):H_{1}, \end{array}$$

where *F*(*s*) is the empirical CDF of the sample. It can be calculated as follows:

(2) $$\begin{array}{c} F\left(s\right)=\frac{1}{n}\overset{n}{\underset{i=1}{\sum }}I\left\{s_{i}\leq s\right\}, \end{array}$$

where *I*{·} is the indicator of event {·}.

There are many types of GOF tests, for instance, Cramer-von Mises (CM), Kolmogorov-Smirnov (KS), AndersonDarling (AD), and Hosmer-Lemeshow (HL) tests. In this paper, we consider two types of GOF tests, CM and KS tests, which can run well with a low number of samples.

 (1) Kolmogorov-Smirnov (KS) test: the KS test, which is based on the empirical CDF of the samples set and the reference CDF, can be calculated according to the largest difference of two distributions as follows:
  (3) $$\begin{array}{c} D_{\text{KS}}=\sup\left\{\left|F\left(s_{i}\right)-F_{0}\left(s_{i}\right)\right|:i=1,\ldots ,n\right\}, \end{array}$$
 where sup⁡{·} is supremum function, which indicates the greatest element of the set. If the sample comes from distribution *F*_0_(*x*), then *D*_KS_ will converge to 0.
 (2) Cramer-von Mises (CM) test: CM test is used for judging the goodness-of-fit of the sample set's CDF *F*(*s*) and reference distribution's CDF *F*_0_(*s*). The test statistic is given by
  (4) $$\begin{array}{c} D_{\text{CM}}=n\overset{+\infty }{\underset{-\infty }{\int }}{\left[F\left(s\right)-F_{0}\left(s\right)\right]}^{2}dF_{0}\left(s\right) \end{array}$$
 and can be approximated as
  (5) $$\begin{array}{c} D_{\text{CM}}=\frac{1}{12n}+\overset{n}{\underset{i=1}{\sum }}{\left[\frac{2i-1}{2n}-F_{0}\left(s_{i}\right)\right]}^{2}. \end{array}$$
 If this value, *D*_CM_, is larger than the threshold, the hypothesis that the sample data come from the reference distribution *F*_0_ can be rejected.

### 2.2. Combination of Evidence in Dempster-Shafer Theory

Dempster-Shafer (DS) theory was first introduced by Demperster and was later extended by Shafer. This is a potentially valuable tool for the evaluation of risk and reliability in engineering applications when it is not possible to obtain a precise measurement from experiments or when knowledge is obtained from expert elicitation. An important aspect of this theory is the combination of evidence obtained from multiple sources and the modeling of conflict between them.

In DS theory [12], a representation of ignorance is provided by assigning a nonzero mass function to hypothesis *m*, also called the basic probability assignment (BPA), and is defined for every hypothesis *A* such that the mass value *m*(*A*) belongs to the interval [0,1] and satisfies the following conditions:

(6) $$\begin{array}{c} m\left(\phi \right)=0,\\ \sum m\left(A\right)=1,\;A\subseteq \Omega , \end{array}$$

where *Ω* is the frame of discernment, which is a fixed set of *q* mutually exclusive and exhaustive elements.

By assigning a nonzero mass in a compound hypothesis, *A* ∪ *B* means that there exists the option to not make a decision between *A* and *B* but to leave the formulation in the *A*∩*B* class. In DS theory, two functions, belief (Bel) and plausibility (Pls), are defined to characterize the uncertainty and support of certain hypotheses. Bel measures the minimum or necessary support, whereas Pls reflects the maximum or potential support for that hypothesis [13]. These two measures, derived from mass values, are defined as a map from a set of hypotheses to interval [0,1] as follows:

(7) $$\begin{array}{c} \text{Bel}\left(A\right)=\underset{B\subseteq A}{\sum }m\left(B\right),\\ \text{Pls}\left(A\right)=\underset{A\cap B\neq 0}{\sum }m\left(B\right). \end{array}$$

The sum of mass functions from different information source, *m*_*j*_  (*j* = 1,2,…*M*), combined with the DS rule is known as the orthogonal sum, which is commutative and associative. The result is a new mass function, *m*(*A*_*k*_) = (*m*_1_ ⊕ *m*_2_ ⊕ ⋯*m*_*d*_)(*A*_*k*_), which incorporates the joints information provided by the sources as follows:

(8) $$\begin{array}{c} m\left(A_{k}\right)=\frac{1}{1-K}\underset{A_{1}\cap A_{2}\cdots A_{d}=A_{k}}{\sum }\left(\underset{1\leq j\leq M}{\prod }m_{j}\left(A_{j}\right)\right),\\ K=\underset{A_{1}\cap A_{2}\cdots A_{d}=\phi }{\sum }\left(\underset{1\leq j\leq M}{\prod }m_{j}\left(A_{j}\right)\right), \end{array}$$

where *K* is the measure of conflict between the different sources and is introduced as a normalization factor.

## 3. The Proposed Secure Cooperative Spectrum Sensing Based on GOF Test

**Figure 1:**
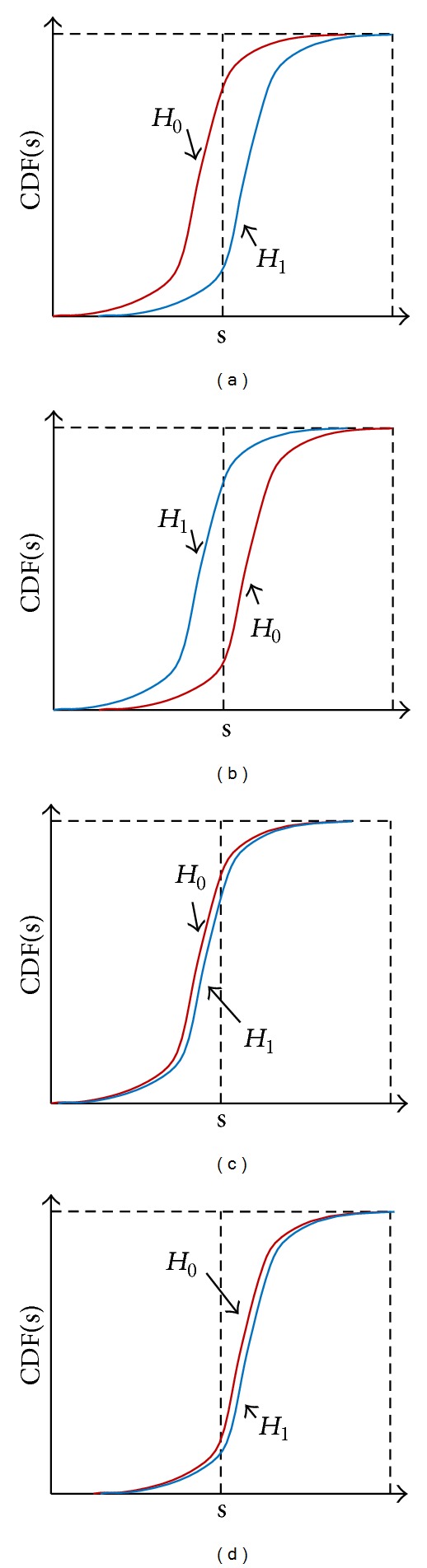
The CDF of received signal energy at CU under absence and presence hypothesis of PU signal for (a) normal CU, (b)* opposite* malicious CU, (c)* always Yes* malicious CU, and (d)* always No* malicious CU.

There is a definite difference between the CDF of received signal energy of normal CU and that of the malicious users as shown in Figure 1. The CDF of the received signal energy of normal CU corresponding to the presence of the PU is always “*under*” that one corresponding to the absence of the PU. On the contrary, the* opposite malicious* CU has the CDF corresponding to the presence of PU to be “*above*” the CDF corresponding to the absence of PU. The* always Yes* and* always No* malicious CUs have a similar CDF corresponding to presence and absence of PU. Due to the difference between CDF of normal and malicious CUs, we utilize GOF test to detect the appearance of malicious users in the network, so that their harmful effect can be rejected out of CSS process. Multi-GOF tests including KS and CM tests will be applied for adaptive robust CSS. The DS theory will be used to combine results of multi-GOF tests.

In this paper, we consider a CR network including *N* CUs who cooperate to sense the signal from a PU. There are *p* < *N* malicious CUs appearing in the network which can be classified as three common types:* always Yes*,* always No,* and* opposite* malicious CUs. All CUs use energy detectors to perform spectrum sensing and send their sensing data to the FC through a control channel. Based on the sensing data obtained from the CUs, the FC makes a global decision concerning the presence or absence of the PU signal by using the proposed data fusion scheme. The proposed scheme has 3 steps as follows.

**Step 1**

All CUs perform spectrum sensing by using energy detection method to determine received signal energy *E*_*j*_ = {*e*_1,*j*_, *e*_2,*j*_,…, *e*_*M*,*j*_}, where *M* is the number of sensing samples that the *j*th CU takes in the sensing interval.

**Step 2**

At the FC, GOF test statistics of each CU will be computed according to hypothesis of the PU as given in (11). After that, BPA and final BPA for current sensing data will be estimated based on the “*reputation level*” of each CU, which is updated from previous sensing interval. Based on final BPA, a global decision rule will be proposed to make global decision about status of PU signal.

**Step 3**

Update “*reputation level*” of each CU according to the global decision.

The detailed description of each step will be given in the following subsections.

### 3.1. Energy Detection

At the sensing interval for the *j*th CU, the local spectrum sensing is to decide between the two following hypotheses:

(9) $$\begin{array}{c} H_{0}:s_{j}\left(k\right)=n_{j}\left(k\right),\\ H_{1}:s_{j}\left(k\right)=h_{j}p\left(k\right)+n_{j}\left(k\right), \end{array}$$

where *H*_0_ and *H*_1_ correspond to the hypothesis of the absence and presence of the PU signal, respectively, *h*_*j*_ denotes the amplitude gain of the channel, *s*(*k*) is the signal transmitted from the PU, *n*_*j*_(*k*) is the additive white Gaussian noise, and *k* is index of sensing sample at each sensing interval.

A received signal energy of a sensing sample, *e*_*k*,*j*_, is given as

(10) $$\begin{array}{c} e_{k,j}=\{\begin{array}{cc} {\left|n_{j}\left(k\right)\right|}^{2}, & H_{0}\\ {\left|h_{j}s\left(k\right)+n_{j}\left(k\right)\right|}^{2}, & H_{1}. \end{array} \end{array}$$

### 3.2. BPA Estimation

The GOF test statistics of the current sensing data *e*_*k*,*j*_  (*k* = 1,…, *M*) of the *j*th CU will be calculated according to each hypothesis of PU signal based on (3) and (5) as follows, respectively:

(11) $$\begin{array}{c} D_{h,j}^{\text{KS}}=\sup\left\{\left|F\left(e_{k,j}\right)-F_{h}\left(e_{k,j}\right)\right|:k=1,2,\ldots ,M\right\},\\ D_{h,j}^{\text{CM}}=\frac{1}{12M}+\overset{M}{\underset{i=1}{\sum }}{\left[\frac{2i-1}{2M}-F_{h}\left(e_{k,j}\right)\right]}^{2}, \end{array}$$

where *h* = {0,1} is index of hypothesis *H*_*h*_ of PU signal, *e*_*k*,*j*_ is the received signal energy of *k*th sensing sample of the *j*th CU, *F*(·) and *F*_*h*_(·) are empirical CDF of observed sensing sample and CDF of *H*_*h*_ hypothesis of PU, and *M* is the number of samples for each sensing interval.

It is noteworthy that normal CU and malicious CU have different characteristics of *D*_1,*j*_^*t*^ and *D*_0,*j*_^*t*^ as shown in Table 1, where *t* indexes types of GOF tests: KS and CM.

Based on the values of *D*_1,*j*_^*t*^ and *D*_0,*j*_^*t*^, we will estimate BPA of current sensing data of each CU and their “*reputation level*” for robust CSS as follows:

(12) $$\begin{array}{c} \Delta_{1,j}^{t}=\frac{D_{0,j}^{t}}{D_{1,j}^{t}+D_{0,j}^{t}}R_{0,j}^{t},\\ \Delta_{0,j}^{t}=\frac{D_{1,j}^{t}}{D_{1,j}^{t}+D_{0,j}^{t}}R_{1,j}^{t}, \end{array}$$

where *R*_*h*,*j*_^*t*^ is “*reputation level*” of the *j*th CU according to hypothesis *h*, and it can be determined based on history observation of the *j*th CU as follows:

(13) $$\begin{array}{c} R_{h,j}^{t}\left(i\right)=\frac{r_{h,j}^{t}\left(i-1\right)}{\sum_{j}^{}r_{h,j}^{t}\left(i-1\right)}, \end{array}$$

where *i* is the index of current sensing interval and *r*_1,*j*_^*t*^(*i* − 1) and *r*_0,*j*_^*t*^(*i* − 1) are updated from the previous sensing interval according to global decision:

(14) $$\begin{array}{c} r_{1,j}^{t}\left(i-1\right)=r_{1,j}^{t}\left(i-2\right)+\left(D_{1,j}^{t}\left(i-1\right)-D_{0,j}^{t}\left(i-1\right)\right), \end{array}$$

(15) $$\begin{array}{c} r_{0,j}^{t}\left(i-1\right)=r_{0,j}^{t}\left(i-2\right)+\left(D_{0,j}^{t}\left(i-1\right)-D_{1,j}^{t}\left(i-1\right)\right). \end{array}$$

By using *r*_1,*j*_^*t*^ and *r*_0,*j*_^*t*^, types of CUs will be easily distinguished. The normal CU has positive value of both *r*_1,*j*_^*t*^ and *r*_0,*j*_^*t*^ that will be increased after updating step. *r*_1,*j*_^*t*^ of* always Yes* and *r*_0,*j*_^*t*^ of* always No* malicious CUs are almost negative and tend to decrease after updating step. On the other hand, both values of opposite CU are negative and have a tendency to decrease. We define “*malicious threshold*” as *ρ* to reject the attack of malicious CR in CSS, so that the CU, which has either *r*_1,*j*_^*t*^ < 0 or *r*_0,*j*_^*t*^ < 0, will be determined as malicious CU. The sensing data of malicious CUs will not be considered to make global decision by giving them *r*_1,*j*_^*t*^ = 0 and *r*_0,*j*_^*t*^ = 0.

The BPA of all CUs will be combined with their reputation levels as

(16) $$\begin{array}{c} \Delta_{1}^{t}=\frac{1}{n_{\Omega }}\underset{j\in \Omega }{\sum }\frac{D_{0,j}^{t}}{D_{1,j}^{t}+D_{0,j}^{t}}R_{0,j}^{t},\\ \Delta_{0}^{t}=\frac{1}{n_{\Omega }}\underset{j\in \Omega }{\sum }\frac{D_{1,j}^{t}}{D_{1,j}^{t}+D_{0,j}^{t}}R_{1,j}^{t}, \end{array}$$

where *Ω* and *n*_*Ω*_ are set of normal CUs and number of members of the set, respectively.

Because the error in estimating Δ_0_^*t*^ and Δ_1_^*t*^,  Δ_0_^*t*^ + Δ_1_^*t*^ can be bigger than 1, we need to normalize those values as

(17) $$\begin{array}{c} \Delta_{0}^{t*}=\frac{\Delta_{0}^{t}}{\Delta_{0}^{t}+\Delta_{1}^{t}},\\ \Delta_{1}^{t*}=\frac{\Delta_{1}^{t}}{\Delta_{0}^{t}+\Delta_{1}^{t}}. \end{array}$$

### 3.3. DS Theory Combination

The DS theory will be used to combine the BPA of both GOF tests according to each hypothesis as follows:

(18) Δ1=Δ1KS∗⊕Δ1CM∗=Δ1KS∗Δ1CM∗1−(Δ1KS∗Δ0CM∗+Δ0KS∗Δ1CM∗),Δ0=Δ0KS∗⊕Δ0CM∗=Δ0KS∗Δ0CM∗1−(Δ1KS∗Δ0CM∗+Δ0KS∗Δ1CM∗).

Finally, the global decision will be made as follows:

(19) $$\begin{array}{c} G=H_{1},\;\text{if}\;\;\frac{\Delta_{1}}{\Delta_{0}}\geq \eta ,\\ G=H_{0},\;\text{otherwise}, \end{array}$$

where *η* is the threshold for global decision.

According to the global decision, *r*_1,*j*_^*t*^ or *r*_0,*j*_^*t*^ will be updated for the next sensing interval as follows, respectively.

- If the global decision is *G*(*i*) = 0, we update *r*_1,*j*_^*t*^(*i*) by using (14).
- Otherwise, we update *r*_0,*j*_^*t*^(*i*) by using (15).

## 4. Simulation Results

In this section, simulation results of the proposed scheme and other soft combination schemes such as maximum gain combination (MGC) and equal gain combination (EGC) are provided. The network is considered in which 5 CUs exist and some of them can be malicious CUs.

**Figure 2:**
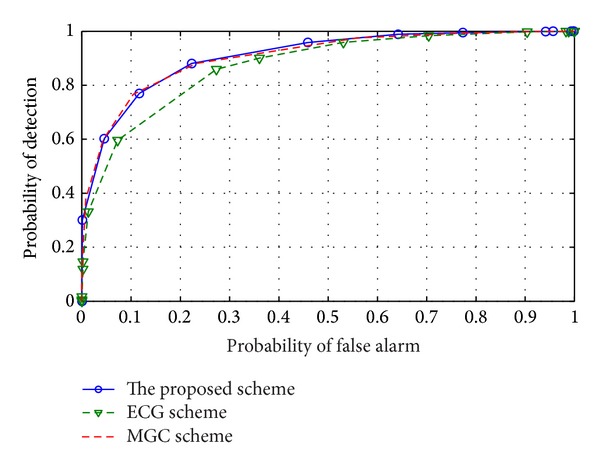
ROC of the proposed scheme and reference schemes when no malicious CU is considered.

In order to verify the reliability of the proposed combination scheme, we perform a simulation without considering malicious CU. The sensing results in Figure 2 show that the proposed scheme can obtain better sensing performance in comparison with EGC scheme and obtain a similar sensing performance to that of the MGC scheme when no malicious CU is considered.

**Figure 3:**
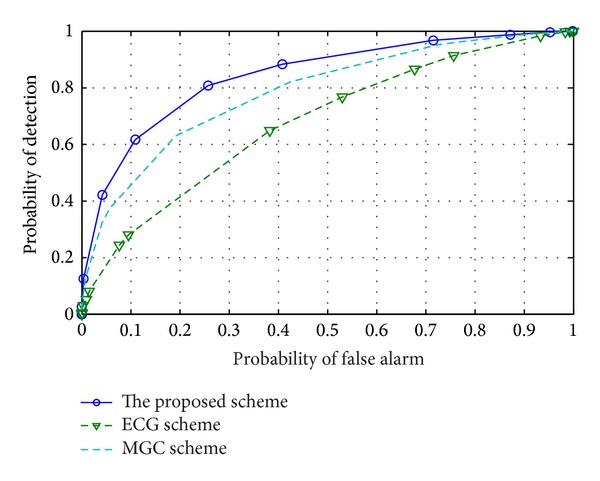
ROC of the proposed scheme and reference schemes when 4* always No* malicious CUs are considered.

**Figure 4:**
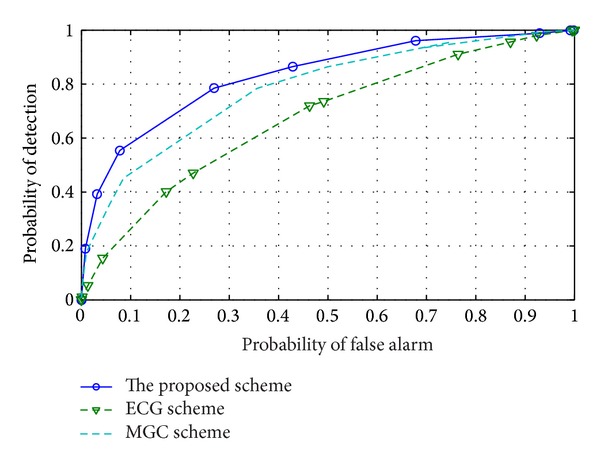
ROC of the proposed scheme and reference schemes when 4* always Yes* malicious CUs are considered.

The robustness of the proposed scheme will be investigated in the network with the appearance of* always No*,* always Yes*, and* opposite* malicious CUs in the network. Figures 3 and 4 show performance of the proposed scheme when 4 CUs are* always No* or* always Yes* malicious CUs among 5 CUs in the network. The results show that the proposed scheme with all CUs can achieve much better sensing performance than that one of the MGC and EGC schemes. This means that, by applying GOF test to CSS, the proposed scheme can detect the presence of those types of malicious CUs and reject their harmful effects to sensing process.

**Figure 5:**
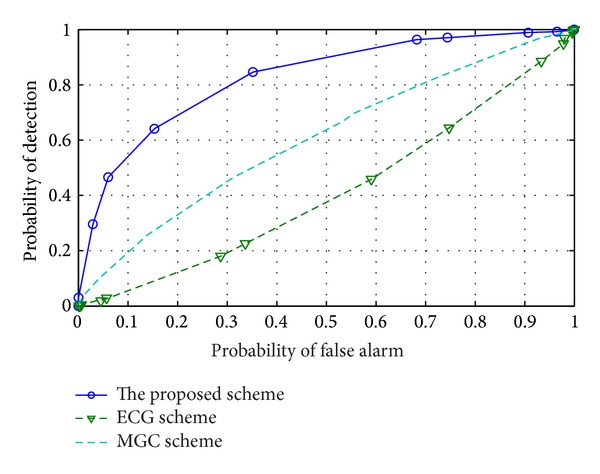
ROC of the proposed scheme and reference schemes when 4* opposite* malicious CUs are considered.

Opposite malicious CU causes the most damage to sensing performance. However, the proposed scheme is expected to protect CSS against this type of malicious CU.  Figure 5 shows the sensing performance of the network when 4 CUs are* opposite* malicious among 5 CUs. MGC and EGC with all CUs provide very low performance due to the attack of* opposite* malicious CU. However, the proposed scheme can defend their attacks and achieve high sensing performance.

## 5. Conclusion

In this paper, multi-GOF tests are proposed to measure the difference between sensing data of normal CU and that of malicious CU. Further, the DS theory is used to combine results of multi-GOF tests. The proposed scheme considers the appearance of the most common types of malicious CU:* always Yes*,* always No,* and* opposite* types. The simulation results prove that the proposed scheme can reject almost harmful effect from those malicious CUs to protect CSS.

## Figures and Tables

**Table 1:** Reputation ranges according to each type of CUs.

Status of PU	Normal CU	*Al* *wa* *ys* Y*es* CU	*Al* *wa* *ys* N*o* CU	*Op* *po* *si* *te* CU
*H*_1_	*D*_0,*j*_^*t*^ ≫ *D*_1,*j*_^*t*^	*D*_0,*j*_^*t*^ ≫ *D*_1,*j*_^*t*^	*D*_0,*j*_^*t*^ ≪ *D*_1,*j*_^*t*^	*D*_0,*j*_^*t*^ ≪ *D*_1,*j*_^*t*^
	*D*_1,*j*_^*t*^ ≈ 0	*D*_1,*j*_^*t*^ ≈ 0	*D*_0,*j*_^*t*^ ≈ 0	*D*_0,*j*_^*t*^ ≈ 0
	*r*_0,*j*_^*t*^(*i*) ≫ 0	*r*_0,*j*_^*t*^(*i*) ≫ 0	*r*_0,*j*_^*t*^(*i*) ≪ 0	*r*_0,*j*_^*t*^(*i*) ≪ 0

*H*_0_	*D*_0,*j*_^*t*^ ≪ *D*_1,*j*_^*t*^	*D*_0,*j*_^*t*^ ≫ *D*_1,*j*_^*t*^	*D*_0,*j*_^*t*^ ≪ *D*_1,*j*_^*t*^	*D*_0,*j*_^*t*^ ≫ *D*_1,*j*_^*t*^
	*D*_0,*j*_^*t*^ ≈ 0	*D*_1,*j*_^*t*^ ≈ 0	*D*_0,*j*_^*t*^ ≈ 0	*D*_1,*j*_^*t*^ ≈ 0
	*r*_1,*j*_^*t*^(*i*) ≫ 0	*r*_1,*j*_^*t*^(*i*) ≪ 0	*r*_1,*j*_^*t*^(*i*) ≫ 0	*r*_1,*j*_^*t*^(*i*) ≪ 0
